# A Bibliometric Review on Gut Microbiome and Alzheimer’s Disease Between 2012 and 2021

**DOI:** 10.3389/fnagi.2022.804177

**Published:** 2022-07-11

**Authors:** Alejandro I. Trejo-Castro, Diego Carrion-Alvarez, Antonio Martinez-Torteya, Claudia Rangel-Escareño

**Affiliations:** ^1^Escuela de Ingeniería y Ciencias, Tecnologico de Monterrey, Monterrey, Mexico; ^2^Departamento de Ciencias Básicas, Universidad de Monterrey, San Pedro Garza García, Mexico; ^3^Departamento de Ingeniería, Universidad de Monterrey, San Pedro Garza García, Mexico; ^4^Escuela de Ingenieria y Ciencias, Tecnologico de Monterrey, Queretaro, Mexico; ^5^Genómica Computacional, Instituto Nacional de Medicina Genomica, Mexico City, Mexico

**Keywords:** Alzheimer’s disease, bibliometric analysis, gut microbiome, gut microbiota, microbiota-gut-brain axis, trend topics

## Abstract

Research on the microbiome has drawn an increasing amount of attention over the past decade. Even more so for its association with disease. Neurodegenerative diseases, such as Alzheimer’s disease (AD) have been a subject of study for a long time with slow success in improving diagnostic accuracy or identifying a possibility for treatment. In this work, we analyze past and current research on microbiome and its positive impact on AD treatment and diagnosis. We present a bibliometric analysis from 2012 to 2021 with data retrieved on September 2, 2021, from the Scopus database. The query includes “Gut AND (Microbiota OR Microbiome) AND Alzheimer*” within the article title, abstract, and keywords for all kinds of documents in the database. Compared with 2016, the number of publications (NPs) on the subject doubled by 2017. Moreover, we observe an exponential growth through 2020, and with the data presented, it is almost certain that it will continue this trend and grow even further in the upcoming years. We identify key journals interested in the subject and discuss the articles with most citations, analyzing trends and topics for future research, such as the ability to diagnose the disease and complement the cognitive test with other clinical biomarkers. According to the test, mild cognitive impairment (MCI) is normally considered an initial stage for AD. This test, combined with the role of the gut microbiome in early stages of the disease, may improve the diagnostic accuracy. Based on our findings, there is emerging evidence that microbiota, perhaps more specifically gut microbiota, plays a key role in the pathogenesis of diseases, such as AD.

## Introduction

In November of 1906, during the 37th Meeting of the Society of Southwest German Psychiatrists in Tubingen, Germany—Alois Alzheimer first described the disease that bears his name (Lage, 2006; Valleix, 2007). A year later, the article entitled “About a peculiar disease of the cerebral cortex” was published (Stelzmann et al., 1995; Strassnig and Ganguli, 2005). Since then, the Alzheimer’s disease (AD) hallmark pathology has been extensively studied, leading to a variety of explanations. One such explanation, and perhaps the most widely accepted to date, is that AD develops as a result of an accumulation of amyloid-β (Aβ) protein fragments outside neurons. These fragments in turn may contribute to cell death by interfering with neuron-to-neuron communication at synapses, and as hyperphosphorylated tau tangles within neurons, blocking the transport of nutrients and other essential molecules inside neurons (Alzheimer’s Association, 2019). Finding an explanation for these pathologies could mean finding a treatment, however, a comprehensive understanding of the etiopathogenesis has not yet been elucidated, directly impacting the failure of several clinical trials (Tobore, 2019). Among several hypotheses that have been proposed to explain the causes of AD, we have: the amyloid hypothesis, calcium homeostasis hypothesis, cholinergic hypothesis, inflammatory hypothesis, lymphatic system hypothesis, metal ion hypothesis, mitochondrial cascade hypothesis, neurovascular hypothesis, and tau propagation hypothesis (Liu et al., 2019). Thus far, they have not yielded a definite treatment or prevention strategy, but they have led to novel approaches that could potentially explain AD.

Similar to the relationship between the gut microbiome and neurodegenerative diseases, the human gut microbiome is the diverse collection of microorganisms (e.g., bacteria, archaea, and fungi) that reside in the gastrointestinal tract (Fung et al., 2017). In fact, there are studies that report that the microbiota may be the etiopathogenesis of amyloidosis in the brains of subjects with AD, since bacterial infection induces Aβ peptide oligometry and the gut microbiota can produce its own peptides (Pistollato et al., 2016).

It is important to keep track of the advances and the contributions from clinicians and basic scientists so that improved diagnosis and better treatments can be developed. In this study, we summarize through a bibliometric approach the most recent, highly cited research, the countries with the highest contribution, and in general, a landscape of published work on the relationship between the gut microbiome and AD during the last decade. Bibliometric analyses are tools that allow for a statistical examination of scientific publications. They mainly help in defining past and future trends on certain research topics (Kokol et al., 2021). They also allow researchers to perform a more in-depth analysis of the collaborations between authors, countries, and understand the impact of scientific publications within the research community (Kutluk and Danis, 2021). While several bibliometric analyses have been published regarding AD (Sorensen, 2009; Song et al., 2015; Serrano-Pozo et al., 2017; Dong et al., 2019; Schilder et al., 2020), none of them tackles the topic of the relationship between the disease and the gut microbiome. Since this is one of the most novel and strong theories in AD pathology development, it is of utter importance to find future trends that could guide researchers to develop new studies.

## Materials and Methods

### Data Source and Search Strategy

Data were retrieved on 2 September 2021, from the Scopus database. The query used was “Gut AND (Microbiota OR Microbiome) AND Alzheimer*” within the article title, its abstract, and its keywords for all the documents in the database. This means that we queried all publications with the words gut, either microbiota or microbiome, and the term Alzheimer’s with any type of ending (e.g., Alzheimer, Alzheimers, and Alzheimer’s).

**FIGURE 1:**
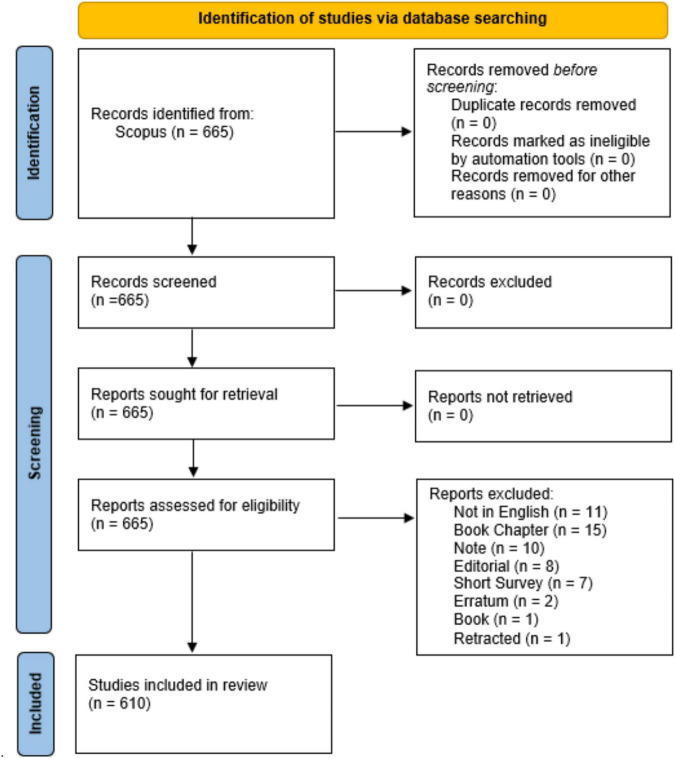
The PRISMA 2020 statement.

Documents were then filtered by language, including only articles written in English, and then by type, considering not only original articles, but also reviews, conference papers, conference reviews, and letters. A PRISMA 2020 flow diagram (Page et al., 2021) depicting the flow of information through the different phases of the analysis can be found in Figure 1. Quantitative and qualitative analyses, such as citation information (e.g., author(s), document title, and year); bibliographical information (e.g., affiliations and publisher); abstract and keywords (e.g., author keywords and index keywords); funding details and other characteristics were extracted using the export document settings in the Scopus database.

### Maps Based on Bibliographical Data

All analyses carried out in this study were performed with the help of the open-source R package bibliometrix (Aria and Cuccurullo, 2017).

## Results

### Main Information

**FIGURE 2:**
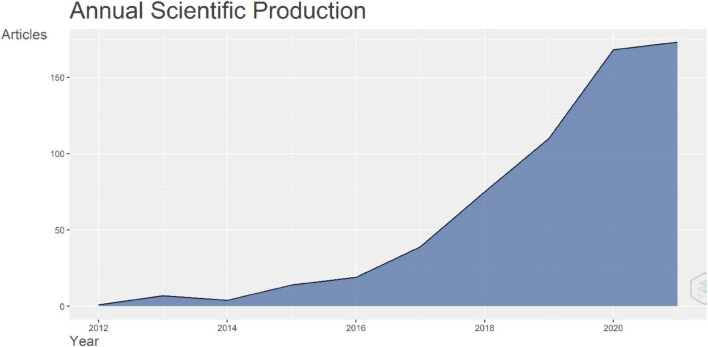
Year-wise distribution of the Scopus documents in 2012–2021.

A total of 610 publications on the relationship between the gut microbiome and AD were found between 2012 and 2021, namely, 318 reviews, 288 original articles, 2 letters, 1 conference paper, and 1 conference review. Descriptive information regarding the collection can be consulted in Table 1 and the year-wise distribution of these publications is shown in Figure 2.

**TABLE 1:** Summary of descriptive information on the collection found from 2012 to 2021.

Main information	Explanation	Count
Documents	Total number of scientific publications	610
Sources	The frequency distribution of sources as journals	312
Author’s keywords	Total number of keywords	1,313
Keywords plus (ID)	Total number of word or phrases that frequently appear in the title of an article’s references	4,839
Authors	Total number of authors	2,764
Authors appearances	The author’s frequency distribution	3,639
Authors of single-authored documents	The number of single authors per articles	39
Authors of multi-authored documents	The number of authors of multi-authored articles	2,725
Authors per document	The average number of authors in each document	4.53
Co-authors per documents	The average number of co-authors in each document	5.97
Single-authored documents	Total number of single-authored documents	41
Documents per author	Total number of documents per author	0.221
Average citations per document	The average number of quotes in each article	29.34
Collaboration index (CI)	Total number of authors of multi-authored articles/total number of multi-authored articles	4.79

**FIGURE 3:**
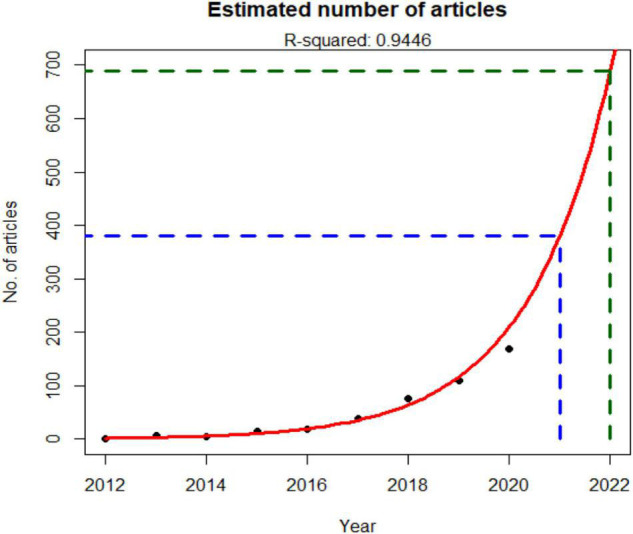
Estimation of publications by year on gut microbiome and AD.

Based on the plot of Figure 2, it is evident that the productivity of this research topic has increased exponentially. It can be expected that the research field would grow further soon. Hence, to test this claim, we fit an exponential regression model on the 2012–2020 data. The results of the regression analysis used to estimate the 2021 and 2022 publications are shown in Figure 3.

According to the regression analysis results, it was estimated that 380 and 688 articles would be published in 2021 and 2022, respectively.

### Scientific Sources

Table 2 shows the top 11 sources that generated most of the publications in the study, specifying the number of publications (NPs), total number of citations (TC), h-index, and the year of the first publication for that source (YFP).

**TABLE 2:** A list of journals with the highest number of publications (NPs) on the subject in our collection.

Source	NP (%)	TC	*h*	YFP
International journal of molecular sciences	25 (4.10)	288	10	2015
Journal of alzheimer’s disease	23 (3.77)	1,052	12	2015
Nutrients	21 (3.44)	602	10	2016
Scientific reports	14 (2.29)	1,461	11	2016
Frontiers in aging neuroscience	13 (2.13)	227	7	2017
Aging	9 (1.48)	136	7	2018
Pharmacological research	9 (1.48)	325	7	2013
Journal of neuroinflammation	8 (1.31)	342	5	2018
Microorganisms	8 (1.31)	509	5	2018
Molecular nutrition and food research	8 (1.31)	87	5	2018
Progress in neuro-psychopharmacology and biological psychiatry	8 (1.31)	101	5	2019

We can see that these journals contribute approximately 25% of the scientific knowledge on the matter. It is important to highlight, however, that even though the International Journal of Molecular Sciences, Journal of Alzheimer’s Disease, and Nutrients have 25, 23, and 21 publications, respectively (+ 3% each), Scientific Reports amassed the most citations (1,461). Further analysis on the primary focus of the most relevant sources revealed that this is indeed an interdisciplinary research field. For instance, the source with the most publications, International Journal of Molecular Sciences, provides an advanced forum for all aspects of molecular research in chemistry. The Journal of Alzheimer’s Disease has principal concerns on the etiology, pathogenesis, epidemiology, genetics, behavior, treatment, and psychology of AD. Nutrients Journal is related to human nutrition with subject areas, such as macronutrients, micronutrients, functional foods, diet-related disorders, and malnutrition. Scientific Reports has a broader scope and publishes original research from all areas of the natural and clinical sciences. As expected, Frontiers in Aging Neuroscience is more focused on the understanding of the underlying mechanisms of central nervous system and aging as well as other age-related neural diseases, but recently, through research topics, which are peer-reviewed article collections around cutting-edge research themes, articles related to the gut microbiome and AD have been published.

### Country Scientific Production

**FIGURE 4:**
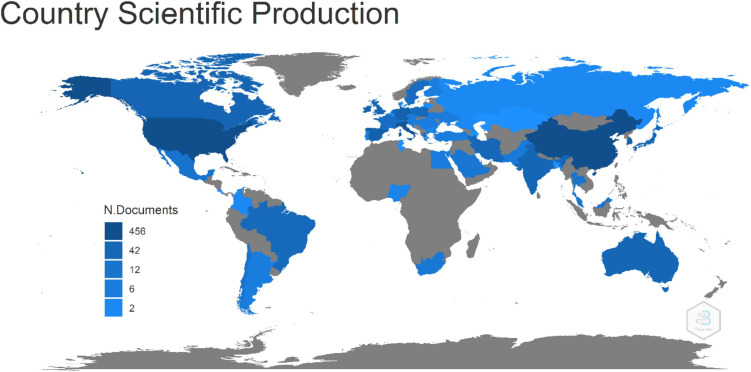
Number of publications per country based on affiliation.

The top 10 countries according to the NPs are listed in Table 3. The worldwide scenario when it comes to AD and gut microbiome is led by three countries: the United States of America (456), China (446), and Italy (226). It is worth noting that percentages do not add up to 100 because, as it will be discussed in the next subsection, a publication could have authors with affiliations from different countries. Figure 4 visually details this metric for all countries.

**TABLE 3:** Number of publications of the most productive countries.

Country	NP (%)
United States of America	456 (74.75)
China	446 (73.11)
Italy	226 (37.05)
Spain	82 (13.44)
South Korea	72 (11.80)
Germany	68 (11.15)
Ireland	65 (10.66)
United Kingdom	56 (9.18)
Canada	51 (8.36)
India	47 (7.70)

**FIGURE 5:**
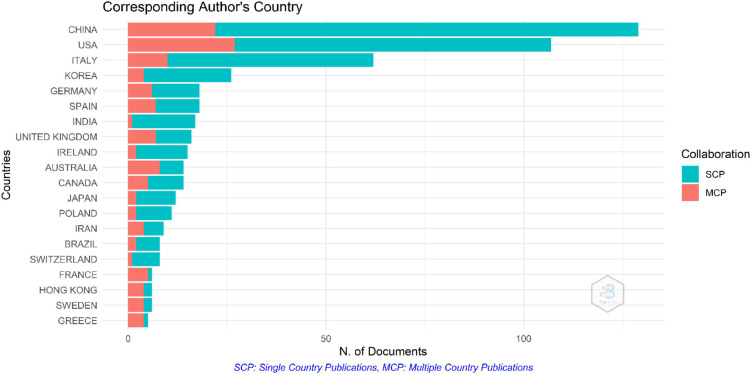
International collaboration by country. Classification goes as multiple country publications (MCPs) in red and at a higher frequency, single country publications (SCPs) in blue.

Several countries played a key role in publishing on the microbiota-gut-brain axis theory for AD. To analyze the international collaboration rate, publications were classified according to the country of affiliation of the corresponding author. Records were classified as either single country publications (SCPs) or multiple country publications (MCPs) and the MCP/NP ratio was calculated. Figure 5 shows the per-country production stratified by the SCP-MCP class.

Countries with the highest international collaboration rate with at least 14 NPs are Australia, the United Kingdom, and Spain, with an MCP/NP ratio of 57.14, 43.75, and 38.89%, respectively. Nonetheless, work needs to done constructing scientific networks cultivating international collaboration in countries, such as India with only 5.88% of their publications classified as MCP or other Latin-American countries that do not even appear in this list.

**FIGURE 6:**
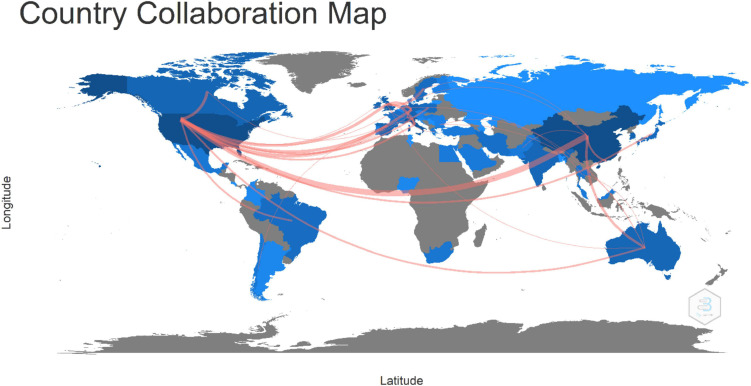
International collaborations among countries. The pink color line represents the connection between countries and its thickness identifies the degree of collaboration. The thicker the line the stronger the collaboration rate.

Among the top four countries in our collection with 610 articles, the major relationships were United States of America-China 18; United States of America-Canada 6; United States of America-Germany 6; and United States of America-United Kingdom 6 as depicted in Figure 6. In this figure, only collaborations with a minimum of three articles were presented. In 2019, at the Lausanne Workshop on AD, the idea of worldwide collaboration was presented. The aim was to develop a global mechanism of action for a better diagnosis and to accelerate research on the drug development that can efficiently treat AD for all people in all places. In 2021, the Davos Alzheimer’s Collaborative launched a global partnership with international organizations from governments and the private sector mobilizing the world against AD. Today, it is recognized that we need a global effort to tackle AD. We would expect to see more pink lines in this map in the following years.

### Citation Analysis

Out of the 610 articles published in the 2012–2021 period on the gut microbiome and AD, the top ten articles that received the most citations are shown in Table 4. It is worth noting that, even though Scientific Reports had the highest number of citations per source, it fell to 4th place in this table.

**TABLE 4:** Publications with the highest number of citations.

Title	First author	Journal	YP	TC
• Interactions between the microbiota, immune and nervous systems in health and disease	Fung, T. C.	Nature Neuroscience	2017	596
• The microbiota-gut-brain axis	Cryan, J. F.	Physiological reviews	2019	468
• What is the healthy gut microbiota composition? A changing ecosystem across age, environment, diet, and diseases	Rinninella, E.	Microorganisms	2019	453
• Gut microbiome alterations in Alzheimer’s disease	Vogt, N. M.	Scientific Reports	2017	439
• Brain-gut interactions in inflammatory bowel disease	Bonaz, B.	Gastroenterology	2013	345
• Association of brain amyloidosis with pro-inflammatory gut bacterial taxa and peripheral inflammation markers in cognitively impaired elderly	Cattaneo, A.	Neurobiology of aging	2017	334
• Microglia in neurodegeneration	Hickman, S.	Nature Neuroscience	2018	320
• Reduction of abeta amyloid pathology in APPPS1 transgenic mice in the absence of gut microbiota	Harach, T.	Scientific Reports	2017	279
• Gut instincts: microbiota as a key regulator of brain development, aging and neurodegeneration	Dinan, T. G.	The Journal of Physiology	2017	256
• The gut microbiota and Alzheimer’s disease	Jiang, C.	Journal of Alzheimer’s Disease	2017	254

*YP stands for year of publication.*

### Trending Topics

**FIGURE 7:**
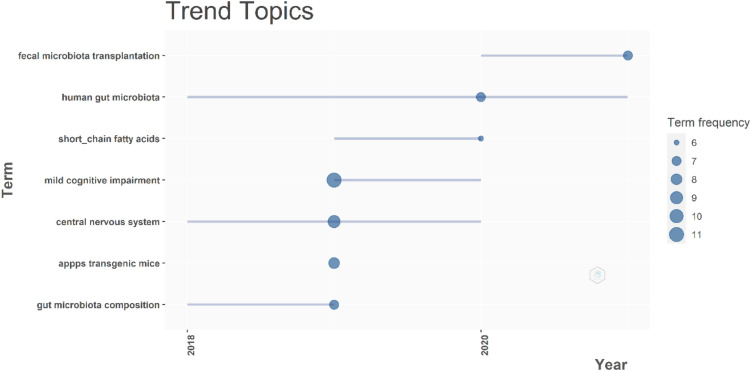
Trending topics. On the *x*-axis, we have the time span in years, on the *y*-axis, the list of trending topics. The size of the circle indicates the frequency of the term, the position of the circle marks the year with most publications on that topic, and the line defines how long that term has been present.

Thus far, we have analyzed the state-of-the-art of published research in AD and microbiota. Another important piece of information is to capture trending topics so future research on AD could focus on emerging priorities for this disease. To uncover trending topics, the titles of the articles in the most recent publications were analyzed through trigrams. Results shown in Figure 7 indicate that trending topics seem to be related to human gut microbiota, short-chain fatty acids, and fecal microbiota transplantation. However, it is also worth mentioning that mild cognitive impairment (MCI), an early stage of memory loss or other cognitive ability loss and a condition that is known to be the prelude to AD, is also trending.

Mild cognitive impairment was a trendy term in 2019 and remains somewhat active, followed by the central nervous system, which does not seem to have the same impact in 2021. There has been an effort to address AD through metabolic changes and even databases, such as the Alzheimer’s Disease Neuroimaging Initiative (ADNI) fully devoted to this approach. However, it is clear that human gut microbiota has today’s attention and that this can get even trendier due to the success of therapies that use fecal microbiota transplantation.

### Conceptual Structure

**FIGURE 8:**
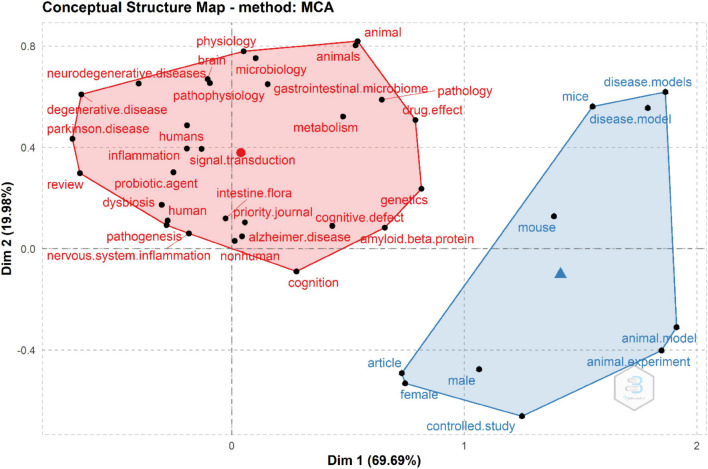
Conceptual structure map. We observe two clusters, the one related with mouse models on the relationship between the gut microbiome and AD, and the other that studies the gut microbiome composition and dysbiosis with or without the impact of other methods in humans like probiotic agents for the restoration of cognition and reduction of amyloid-β fragments.

The conceptual structure represents relations among concepts or words in a set of publications. It is what scientists talk about, the main themes and trends. We performed a factorial analysis—a data reduction technique that serves as a filter of terms that are mostly redundant or of low frequency on the KeyWords Plus. Reduction is achieved by selecting terms that represent a linear combination of other redundant terms through multiple correspondence analysis. Figure 8 is shown below.

Here, the proximity between words is because a large proportion of articles use them together, and the origin of the map represents the average position of all column profiles and therefore represents the center of the research field (Cuccurullo et al., 2016).

## Discussion

Research on microbiome has drawn an increasing amount of attention over the past decade. Even more so for its association with disease. On the other hand, AD has been a subject of study for a longer time with a lack of success in improving diagnostic accuracy or a possibility for treatment. Based on our findings, there is emerging evidence that microbiome—perhaps more specifically the gut microbiome—is a key player in the pathogenesis of diseases, such as AD. In the case of microbiome and AD, a significant number of scientific publications have been published lately. From a selection of 610 scientific contributions in the fields in a Scopus search, we found 52.13% review articles, 47.21% original research articles, and an almost a negligible percentage of conference papers. Compared with 2016, we identified an important increase in 2017 only to double the number of papers in 1 year. Nevertheless, it was just a starting point that led to an exponential growth by 2020 and, with the data presented, it is almost certain that it will continue this trend and grow even further in the upcoming years. Our projections estimate that 2021 will end with almost 400 papers in the subject while this number will almost get to 700 in 2022.

We also identified key journals in the subject. The International Journal of Molecular Sciences, Q1 and 2020 JCR impact factor of 5.923 and open access covered 4.10% of publications from our search. In terms of citations, Scientific Reports Q1, 2020 JCR impact factor of 4.379, open access, led with 1,461 followed by Journal of Alzheimer’s Disease Q1, 2020 JCR impact factor of 4.472 with 1,052. From the top 11 journals, Pharmacological Research is the oldest, with a publication on the matter being 8 years of existence while the other account for less than 5 years. Furthermore, Progress in Neuro-Psychopharmacology and Biological Psychiatry is the newest of these journals with only 2 years of discussion on the topic. The higher h-index among the journals corresponded to the Journal of Alzheimer’s Disease (12), followed by Scientific Reports (11), and a tie in third place between International Journal of Molecular Sciences and Nutrients with an h-index of 10 each. Somehow without a surprise, we found that the United States of America and China are the leaders in terms of NPs with 456 and 446 total articles, respectively. Italy has the third place with 226 publications, counts onward an average of approximately 63 for the following seven countries.

It is encouraging to discover that the two most cited papers (587 and 450 cites, respectively) hit right on target “Interactions between the microbiota, immune and nervous systems in health and disease” published in Nature Neuroscience, and “The microbiota-gut-brain axis” in Physiological Reviews. Both have come a long way from the 5th more cited article “Brain-gut interactions in inflammatory bowel disease” published in 2013. While Nature Neuroscience published the most cited paper to date, it is Scientific Reports the journal that amassed most citations with two of the most cited papers in the topic (4th and 8th). We hypothesize that this might be because the latter is fully open access while the former is still in a transition mode but not entirely open access.

While the tendency to publish in this theme will certainly increase in the upcoming years, a bibliometric analysis allows us to explore trending topics (Figure 7) that we can find one of the most interesting is “short-chain fatty acids.” This corresponds to some novel theories that state the role of these molecules in the development of AD while they are derived as microbial metabolites due to gut dysbiosis (Marizzoni et al., 2020). This trend has just gained interest since 2019.

A major goal in AD research is to be able to diagnose the disease beyond the cognitive test complementing it with other clinical biomarkers. MCI is normally considered an initial stage for AD. This test, in conjunction with the role of the gut microbiome in early stages of the disease, has the potential to improve the diagnostic accuracy.

Another trend that will most likely increase because its direct association to treatment is “fecal microbiota transplantation.” This technique initially developed to treat patients with *Clostridium difficile* infection (CDI) after prolonged antibiotics schemes (Cammarota et al., 2014), however, its use to control gut microbiome dysbiosis has been increasing and it is being examined as a possible treatment for pathologies, such as AD or Parkinson in *in vivo* models (Sun et al., 2019). Furthermore, a case reported by Hazan in 2020 showed cognition improvement in a patients with AD-CDI after a fecal transplant, increasing the Mini-Mental State Examination (MMSE) score from 20 to 29 points within 6 months (Hazan, 2020). Another case was reported this year with similar findings, a 90-year-old woman with AD and severe CDI improved after the fecal transplant; subsequently, this increase in cognitive function was associated with certain bacterial genera, such as *Bacteroides, Bacteroidia, Tannerellaceae*, and *Actinobacteria.* Moreover, the short-chain fatty acids were found to be significantly different between before and after fecal microbiota transplant (Park et al., 2021). Other researchers have characterized the gut microbiome of patients with AD through fecal samples, finding that reductions in *Faecalibacterium* and increases in *Bifidobacterium* were significantly correlated with clinical indicators of the AD (Ling et al., 2021).

Animal models have been used to understand the microbiome role in AD. We observe that in “appps transgenic mice” trend. However, we can soon expect more human-oriented research, particularly in microbiota research. The structure of knowledge analysis shows two main groups. On the one hand, there are murine models of AD, which mainly presents the basic science research focused on transgenic animals and the development of new models, characterized by the words mice, animals, and model. On the other hand, a cluster focused on the role of dysbiosis in the disease within human population. Not only in the role of the microbiota in neurodegenerative diseases, such as AD or Parkinson, but also on the role of probiotics and prebiotics in the management of the disease.

Research overall has been moving toward a better understanding of the microbiome’s role in human health. The gut-brain axis has been followed by other terms, such as the most recently described gut-skin axis, which explores the links between the gut composition and different skin processes, such as keratinization and modulation of the cutaneous immune response (Salem et al., 2018). This new field has motivated researchers in several disciplines to study the microbiome and its effects allowing us to steadily comprehend the role the microbiota plays and how to use them to our benefit. AD is not the exception, by September 14, 2021, we found 14 clinical trials registered in clinicaltrials.gov that explore this relationship. Currently, nine out of those are still recruiting participants, three have been completed, one has been terminated, and another is reported as “unknown.” While this is a still a small amount, it is indeed the start of the novel research in this area. While some steps have already been taken and some degree of characterization has been achieved, there is still some way to research (Rinninella et al., 2019).

Some gut microorganisms have already been hypothesized to play a role in the pathogenesis of AD, such as *Lactobacillus spp.* beneficial to brain functions leading to an optimistic therapy to improve cognitive function (John et al., 2021). Other researchers correlate periodontal hygiene and microbial composition in the mouth to the development of the disease, suggesting a possible therapeutic in the future (Ryder and Xenoudi, 2021). Metabolic diseases have already addressed the role of prebiotics as modifiable factors in insulin resistance diseases with products, such as grenade juice in some patients with the presence of enzymes as urolithin A (Li et al., 2015). The use of non-antibiotic medications has also been described as a gut microbiome modulator; it has even been tried as a novel therapeutic agent. Research within this subject has shown that atorvastatin, a drug from the statins family, can be used to treat hypercholesterolemia and prevent cardiac disease by modifying the gut microbe composition diminishing proinflammatory species and increasing anti-inflammation ones, such as *Akkermansia muciniphila* and *Faecalibacterium prausnitzii* (Khan et al., 2018). Dietary interventions and the supplementation with probiotics of the studied bacteria or other commensal organisms are known to increase the target inside the gut and have been widely explored in metabolic diseases, cancer (Huang et al., 2019), and neurological diseases, such as multiple sclerosis (Mirza et al., 2020) and epilepsy (Dooling and Costa-Mattioli, 2018).

## Conclusion

The role of microorganisms in non-infectious diseases has been growing in recent years. A case of pathogenesis of virus in Epstein-Barr or bacteria, such as *Helicobacter pylori* and its association to gastric cancer and gastritis are only two examples. We are now entering an era in which the question is no longer whether or not the microbiome has an impact on human health. Investigations in AD are beginning to open into this field, and we are at the start of an exponential growth in knowledge generation as well as hope for accurate diagnosis and treatment for AD. This work presents a trending analysis of emerging topics addressing AD. Some will not only remain in the upcoming years but may become the future for new drugs and a step forward toward precision medicine. Microbiota composition varies largely from person to person and even more so from population to population, mainly because of diet and lifestyles. Hence, studies focusing merely on microbiota composition may not be sufficient. There should be an increase in longitudinal studies with follow-ups that evaluate treatments and response and in before-and-after alpha and beta diversities of microbes. Research into whether dysbiosis can be treated with fecal transplantation, the use of prebiotics or probiotics. We know AD is highly correlated to metabolism, and in turn, metabolism is driven primarily by the microbiota. We expect this work not only presents a summary of what has been done so far but it will serve as an insight into the future possibilities of AD treatment.

## Data Availability Statement

The original contributions presented in this study are included in the article/supplementary material, further inquiries can be directed to the corresponding author/s.

## Author Contributions

AT-C, CR-E, and DC-A: conceptualization, investigation, writing original draft preparation, and methodology. AT-C: software and data curation. AT-C, DC-A, and AM-T: validation. AT-C, DC-A, AM-T, and CR-E: formal analysis. AT-C and CR-E: resources. AM-T and CR-E: writing-review and editing. AT-C and AM-T: visualization. CR-E: supervision, project administration, and funding acquisition. All authors have read and agreed to the published version of the manuscript.

## Conflict of Interest

The authors declare that the research was conducted in the absence of any commercial or financial relationships that could be construed as a potential conflict of interest.

## Publisher’s Note

All claims expressed in this article are solely those of the authors and do not necessarily represent those of their affiliated organizations, or those of the publisher, the editors and the reviewers. Any product that may be evaluated in this article, or claim that may be made by its manufacturer, is not guaranteed or endorsed by the publisher.
